# Safety, Tolerability, and Proof-Of-Concept Study of OKV-119, a Novel Exenatide Long-Term Drug Delivery System, in Healthy Cats

**DOI:** 10.3389/fvets.2021.661546

**Published:** 2021-05-11

**Authors:** Michael Klotsman, Christopher A. Adin, Wayne H. Anderson, Chen Gilor

**Affiliations:** ^1^Okava Pharmaceuticals, San Francisco, CA, United States; ^2^Department of Small Animal Clinical Sciences, College of Veterinary Medicine, University of Florida, Gainesville, FL, United States; ^3^Pulmonary and Critical Care Medicine, University of North Carolina at Chapel Hill, Chapel Hill, NC, United States

**Keywords:** diabetes, obesity, adherence, compliance, exenatide, feline, glucagon-like peptide-1, glucagon-like peptide-1 receptor agonist

## Abstract

**Background:** Glucagon-like peptide-1 (GLP-1) is an incretin hormone that plays an important role in glucose homeostasis and food intake. In people, GLP-1 receptor agonists (GLP-1RAs) are commonly used for the treatment of type 2 diabetes mellitus (DM) and obesity; however, non-adherence to injectable medications is common. OKV-119 is an investigational drug delivery system intended for subdermal implantation and delivery of the GLP-1RA exenatide for up to 6 months.

**Hypothesis/Objectives:** Develop protocols for the subcutaneous (SC) insertion and removal of OKV-119 and to evaluate its tolerability, *in vivo* drug-releasing characteristics, and weight-loss effects in cats.

**Animals:** Two cadaveric and 19 purpose-bred cats.

**Methods:** In cadavers, OKV-119 insertion protocol and imaging were performed at three SC locations. The safety and tolerability of OKV-119 implants were assessed in a small (*n* = 4 cats) 62-day study. Weekly plasma exenatide concentrations and body weight were measured in a 42-day proof-of-concept study designed to evaluate OKV-119 prototypes implanted in cats (*n* = 15).

**Results:** In anesthetized cats, the duration of insertion and removal procedures was 1–2 min. OKV-119 was easily identified on radiographs, and well-tolerated without any apparent implant site reactions. Following implantation, exanatide plasma concentrations were observed for up to 35 days. Plasma exenatide concentrations were correlated to weight loss.

**Conclusion and clinical importance:** Our findings suggest that OKV-119 could be easily inserted and removed during a routine clinic visit and can be used to safely and effectively deliver exenatide. Future studies of OKV-119, configured to release exenatide for a longer extended months-long duration, are warranted to determine whether the combination of metabolic improvements and beneficial weight-loss, coupled with minimal impact on pet-owner's lifestyle, lead to improved outcomes for obese cats and feline DM patients.

## Introduction

Glucagon-like peptide-1 (GLP-1), an important incretin hormone that is secreted from the intestines during a meal and participates in the regulation of systemic metabolism, food intake, gastrointestinal motility, and more (1). GLP-1 exerts its main regulatory effect by stimulating glucose-dependent insulin secretion from pancreatic islets (1). GLP-1 receptor agonists (GLP-1RAs) are now a cornerstone treatment of type 2 diabetes mellitus (DM) in human medicine (2), with exenatide being the first-in-class GLP-1RA to be approved (3). GLP-1RAs have also been shown to beneficially reduce body weight in non-diabetic obese people, without causing hypoglycemia or other serious adverse events (AEs) (4–7). Weight-reducing effects are partly attributed to GLP-1 inhibitory effects on gastric emptying, postprandial glucagon release, and stimulation of hypothalamic satiety centers (8).

Although limited, data in cats demonstrate that GLP-1RAs generally have similar effects to those seen in human patients and might be useful in treating feline DM (9–16). Feline obesity, formally classified as a disease, is also major health concern (17). Obesity in cats is associated with numerous cormobidities including DM, osteoarthritis, impaired respiratory function, and certain types of neoplasia (18, 19). Given the clinical challenge of managing feline obesity (19, 20), it is equally intriguing to determine whether, like in human medicine, GLP-1RAs administered to non-diabetic, clinically obese cats can be used to treat obesity and prevent, or delay, the onset of DM and other untoward health outcomes.

In human medicine, non-adherence to antidiabetic medications is common and results in poor long-term glycemic control and the development of diabetic complications (21–23). Medication non-adherence and non-persistence, or discontinuation, have been shown to be particularly common among those taking injectable antidiabetic medications (23). A research survey evaluating injectable insulin has shown that human patients consider injections to be a serious burden and have a negative impact on quality of life (24). Not surprisingly, human studies in real-world settings report low adherence of only 30–50% for injectable GLP-1RAs (25, 26). While adherence and persistence have not been studied in people giving injectable antidiabetic medications to cats, it is anticipated that the problems noted in human patients will only be compounded by the physical and emotional challenges of performing repeated injections in cats. Treatment strategies that account for real-world constraints and limit pet owner responsibilities (e.g., eliminate at-home glucose monitoring) may help to improve long-term outcomes (27).

Long-term extended-release (ER) technologies that minimize injection burden have been shown to be well-tolerated and improve self-reported quality of life in human diabetic patients (28, 29). Given the known challenges of administering chronic medicines to feline patients (30, 31), the benefits of a “hassle-free” long-term drug delivery system may be magnified in cats. Moreover, due to the inherently short half-life and rapid clearance of exenatide in cats (10, 14), an ER preparation may mitigate undesirable side effects associated with “peak-and-valley” pharmacokinetic profiles (32).

**Figure 1:**
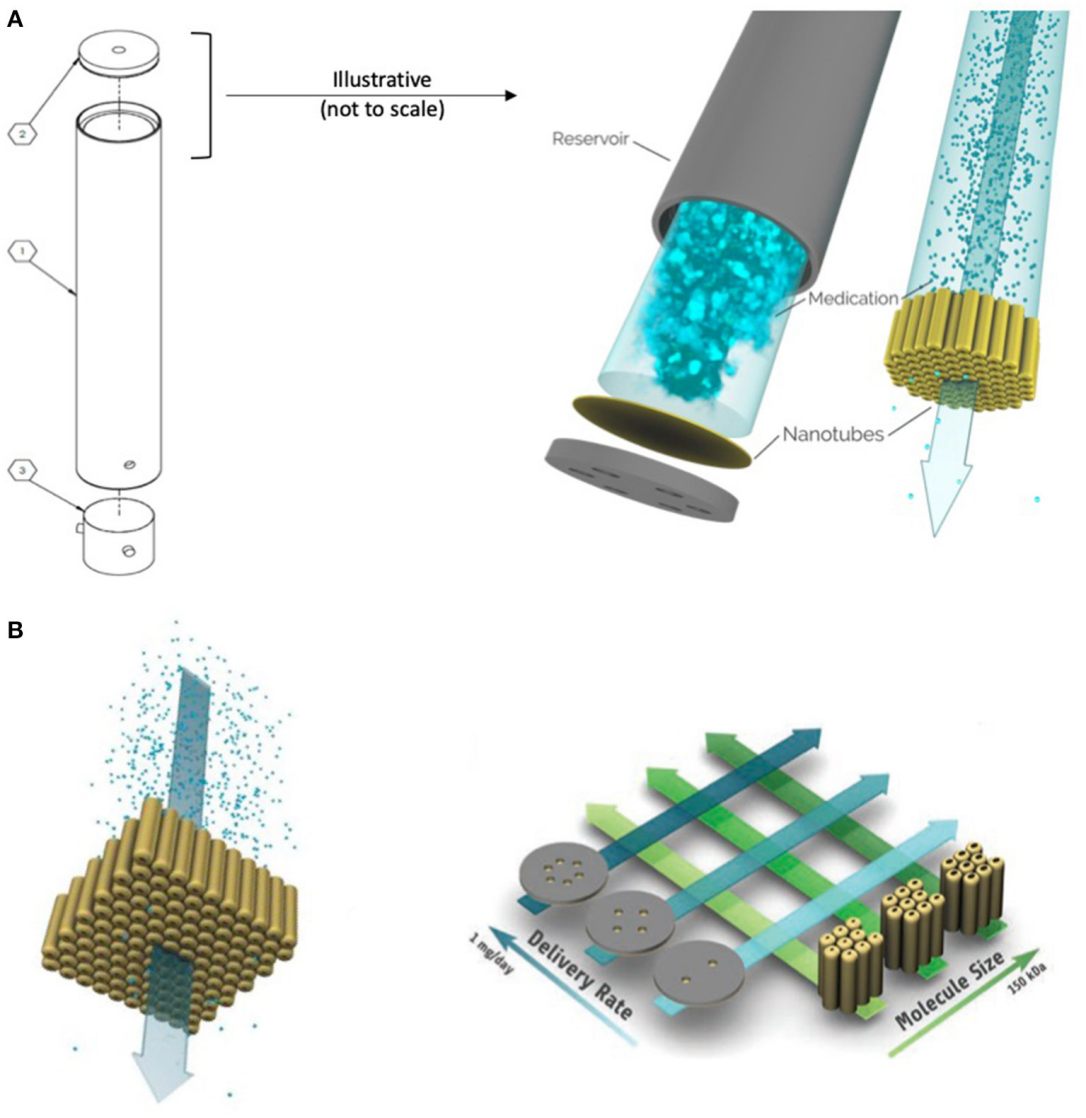
Schematic of the OKV-119 components, titanium reservoir (part 1), NanoPortal membrane (part 2) and silicone septum (part 3) **(A)**. This drug delivery system measures 2 mm by 21.5 mm. Exenatide is delivered from solution inside a reservoir at a rate that remains constant until the drug is nearly fully depleted. The rate of drug release is controlled by a nanoporous membrane composed of biocompatible titanium oxide (titania) nanotubes and is fabricated affixed to a biocompatible titanium substrate. Target delivery rates can be achieved by adjusting the number of accessible nanotubes (i.e., the number of windows is correlated to release rate) and the size of the nanopores (i.e., larger pores have faster release rates) **(B)**. [figures reproduced from Fischer et al. (33) and Fischeret al. (34)].

Herein, we report our initial experiences with an investigational drug delivery system (OKV-119; Figure 1) that will be designed to produce constant, months-long delivery of exenatide. Our first objective was to develop protocols for the subcutaneous insertion and removal of OKV-119 in cats. The second objective was to evaluate the safety of supratherapeutic exenatide doses and to assess the tolerability of the implant in healthy purpose-bred cats. The third objective was to concept test the *in vivo* drug releasing properties of OKV-119 prototypes over a 42-day period. Therefore, weekly exenatide plasma concentrations and corresponding weight-loss effects were measured in healthy cats implanted with OKV-119 prototype systems.

## Materials and Methods

### Investigational Drug-Delivery System

The overall design and primary working principle of the OKV-119 systems used herein were based on the prototype depicted previously (33, 34). Made from titanium and titania, the implant is composed of a cylindrical drug reservoir with the NanoPortal™ membrane attached at one end and a molded silicone septum closure at the other end (Figure 1A). OKV-119 is optimized for biocompatibility; once fully assembled, the only material in contact with the body is titanium oxide and silicone. All OKV-119 components, excipients, and materials have been used in FDA approved products.

The rate of exenatide released from the OKV-119 implant is controlled by adjusting pore size and the exposed number of nanotubes (Figure 1B) (33, 34). Smaller nanotube pore size (generated by increased atomic layer deposition) and fewer exposed nanotubes elicit slower drug release rates. OKV-119 prototypes used in the present study released a peak of ~50–100 μg exenatide per day.

### Insertion and Removal Procedures

An initial study was performed in two cat cadavers to develop insertion and removal techniques of OKV-119 and to evaluate radiopacity and echogenicity of the implant following implantation (University of Florida Institutional Animal Care and Use Committee protocol #201910973). OKV-119 implants were placed in three subcutaneous locations in each cat: the medial brachium, dorsal lumbar, and lateral crus. Imaging was performed using digital radiography and ultrasound of each location to determine the most suitable modality for locating the implant before removal in live animals. Implantation sites were compared for ease of insertion and removal in this species.

In a subsequent safety and tolerability study, cats (*n* = 4) were anesthetized with 2 mg/Kg xylazine (20 mg/mL) and 1 mg/Kg ketamine (100 mg/mL) followed by administration of inhaled 4% isoflurane. A 4 × 4 cm area was clipped and aseptically prepared on the dorsal lumbar area. A 12 g needle was loaded with OKV-119 through its beveled tip until it was entirely within its lumen. A K-wire was then inserted into the opposite side of the needle while tilting the needle upwards and avoiding expulsion of the implant through the beveled tip of the needle. Next, the skin was incised (1 mm) with a scalpel. The skin was held with forceps close to the incision and the 12 g needle (and the and K-wire in it) was inserted about 3 cm into the subcutaneous tissue through the incision. The needle was then retracted over the K-wire while stabilizing the K-wire, causing the OKV-119 implant to be pushed out of the needle and into the space that was created by the retracted needle. The K-wire was removed, the skin edges were apposed, and the incision closed with a drop of tissue glue. Finally, the anesthesia was discontinued and the cat was monitored until fully awake.

Lastly, in a OKV-119 proof-of-concept study, cats (*n* = 15) were anesthetized and the implant site was prepared as described above. The skin was incised (2–3 mm) with a scalpel. The scalpel was then used to gently create a small subcutaneous cavity. The OKV-119 implant was inserted using Brown-Adson tissue forceps to hold the implant at its center. After insertion, the skin edges were apposed, and the incision closed with a drop of tissue glue.

To extract the implant, cats were sedated with 2 mg/Kg xylazine and 1 mg/Kg ketamine and a 4 × 4 cm area was clipped and aseptically prepared over the implant. Using Brown-Adson tissue forceps to hold the implant at its center, the tip of the implant was pushed toward the skin incision. The tissue surrounding the tip of the implant was dissected using a scalpel until it was exposed. The implant was then pulled out and the skin incision closed with a drop of tissue glue (Videos of the insertion and removal procedures are provided online).

### Safety and Tolerability Study

The safety and tolerability of OKV-119 was initially assessed over an 8-week period. In this part of the study, prototype OKV-119 implants configured to release supratherapeutic exenatide doses were inserted subcutaneously in healthy, purpose-bred, domestic short hair cats in the lateral crus (*n* = 2) and dorsal lumbar (*n* = 2).

Cats were considered healthy at time of enrollment based on physical examination. They were acclimated for 7 days prior to treatment in the room where they were to be housed during the study. During the course of the study, cats were housed separately in pens in compliance with current recommendations for the Guide for the Care and Use of Laboratory Animals and under the standard operating procedures of the testing facility. Cats were fed a standardized pelleted feline diet *ad libitum*, with fresh feed provided once daily in the morning. The amount of food provided and consumed was recorded throughout the study. Water was supplied *ad libitum*. Daily physical exams were performed. Body weight measurements and exenatide plasma samples were taken weekly during the course of the study. Serum biochemistry and complete blood counts were measured at baseline and on the last study day. The study protocol was approved by the AAALAC accredited test facility (IACUC protocol #TH200118).

### OKV-119 Drug Release Proof-Of-Concept Study

A 6-week study, in which weekly exenatide plasma concentrations were measured, was performed to evaluate OKV-119 implants configured to release exenatide at three different rates. Cats in Group 1 were implanted with OKV-119 configured with the largest nanopores (i.e., fastest release rate), while the implants used for Group 3 were configured using the thickest ALD profile (i.e., slowest release rate). The selection of these prototype OKV-119 configurations was informed by prior studies performed in rats (unpublished data).

This laboratory-based study in healthy purpose-bred domestic short hair cats was conducted under conditions that were nearly identical to the safety and tolerability study detailed above. Briefly, acclimated cats were randomized to Group 1 (*n* = 3), Group 2 (*n* = 6), or Group 3 (*n* = 6). Cats were individually housed and monitored throughout the study. Body weight and exenatide plasma measurements were collected weekly. Implants were removed from the cats on a pre-determined weekly schedule (Table 1) to evaluate the integrity of the drug delivery system. The study protocol was approved by the AAALAC accredited test facility (IACUC protocol #TH200118).

**Table 1:** Exenatide plasma concentrations (ng/ml) in cats after subcutaneous implantation of OKV-119 in Group 1 (*n* = 3, largest NanoPores with the fastest release), Group 2 (*n* = 6, medium thickness ALD), and Group 3 (*n* = 6, smallest NanoPores with a slower release).

	**Age (months)**	**Gender**	**Day 7**	**Day 14**	**Day 21**	**Day 28**	**Day 35**	**Day 42**
**Group 1**								
Cat 1	47.0	Male	3.02	2.55	2.71	4.87^a^		
Cat 2	47.0	Male	2.67	3.86^a^				
Cat 3	66.5	Male	1.65^a^					
*Median*			*2.67*	*3.21*				
**Group 2**								
Cat 1	47.0	Male	2.78	2.42	2.62	1.04	0.732	0^a^
Cat 2	47.0	Male	4.33	2.02	0.30	1.11	0.961	0.575^a^
Cat 3	67.0	Male	0.332	0.315	0.268	1.04^a^		
Cat 4	66.5	Male	1.94	1.73^a^				
Cat 5	66.5	Male	2.3	12.9^b^	21.8^a^^,^^b^			
Cat 6	40.0	Female	4.2^a^					
*Median*			*2.54*	*2.02*	*1.444*	*1.04*	*0.8465*	*0.2875*
**Group 3**								
Cat 1	47.0	Male	2.83	1.37	2.71	4.14	1.47	0.266^a^
Cat 2	66.5	Male	0.657^a^					
Cat 3	47.0	Male	0.471	0.242				
Cat 4	22.0	Male	0	0.983	1.05	6.36	0.433	0^a^
Cat 5	40.0	Female	0.603	1.3	1.13	0.78^a^		
Cat 6	21.5	Male	0	0.206	0.3	0.57	1.02	0.613^a^
*Median*			*0.54*	*0.98*	*1.09*	*2.46*	*1.02*	*0.27*

a *OKV-119 implants were removed on a pre-determined weekly schedule*.

b *Outliers were excluded from the analysis. Shaded areas correspond to time-points after which the OKV-119 implants were removed*.

### Blood Sampling and Exenatide Plasma Concentration Assay

Blood samples were collected once prior to implantation (Day 0) and then weekly for the duration of each study. In the proof-of-concept study, all samples were collected after an overnight fast.

Blood samples were collected from the jugular vein and placed in pre-chilled EDTA tubes that were immediately placed on ice. Samples were then centrifuged (4°C, 3,000 RPM, 15 min), plasma was collected, divided into 4 aliquots and stored at −80°C until analysis.

A qualified LC-MS/MS method for the determination of exenatide concentrations in feline plasma was developed at an independent laboratory^1^. Assay range was 0.5–100 ng/ml. Linear regression for expected vs. observed results in serial dilutions in this range were *R*^2^ = 0.997 (*P* < 0.0001), slope = 1.0 ± 0.02 (CI = 0.96–1.07) and a Y intercept = −0.73 ± 0.91. Recovery of quality control material was 106.6% at 3 ng/mL and 103.0% at 75 ng/mL. When plasma exenatide concentrations were below the level of detection (0.5 ng/mL), a value of 0 ng/mL was assigned.

### Statistical Analysis

Statistical analysis was performed using a commercially available computer software^2^. Clinical parameters, AEs, exanatide plasma concentrations, and laboratory parameters were summarized descriptively. Data are presented as median [range]. Outliers were identified by visualization of the plasma concentration data and removed the analysis. Data were assessed for normality using Shapiro-Wilk. Pearson's r was used to assess the correlation between weekly percent change in body weight and exenatide concentration measurements in the safety and tolerability study. In the subsequent OKV-119 proof-of-concept study, weekly percent change in body weight and exenatide concentration measurements were not normally distributed and their correlation was assessed by the Spearman test. Outliers were identified by the Rout test and removed the correlation analysis. Significance was set at *p* ≤ 0.05.

## Results

**Figure 2:**
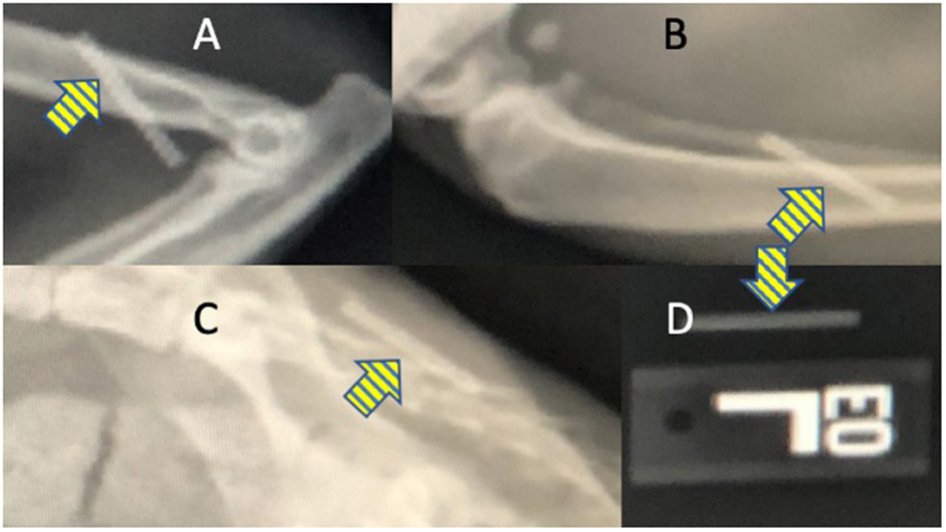
Radiographic images show that the OKV-119 implant is radiopaque (yellow arrows) and can be identified after implantation in cats at the **(A)** medial brachium, **(B)** lateral crus, and **(C)** dorsal lumbar regions. For comparison, the appearance of the OKV-119 implant before implantation is shown in **(D)**.

After insertion in the SC space, the implant is palpable. To prepare for the possibility of migration over time and the need to locate the implant anywhere in the body, radiographs were obtained after inserting implants at different locations in 2 cat cadavers. The implant is radiopaque and was easily identified on radiographs at all insertion sites including when overlaid by bone (Figure 2). The echogenicity of the implant was similar to surrounding SC tissue during ultrasound examination. The tubular shape of the implant could be identified when the location of the device was known to the ultrasonographer but it was estimated that locating a implant that migrated from the site of insertion would be challenging using this modality. Based on the ease of access and low risk for damage to vital structures, the dorsal lumbar and lateral crus sites were considered most suitable for future studies in live animals.

In anesthetized cats, the duration of insertion and removal procedures was 1–2 min.

### Tolerability and Safety

**Figure 3:**
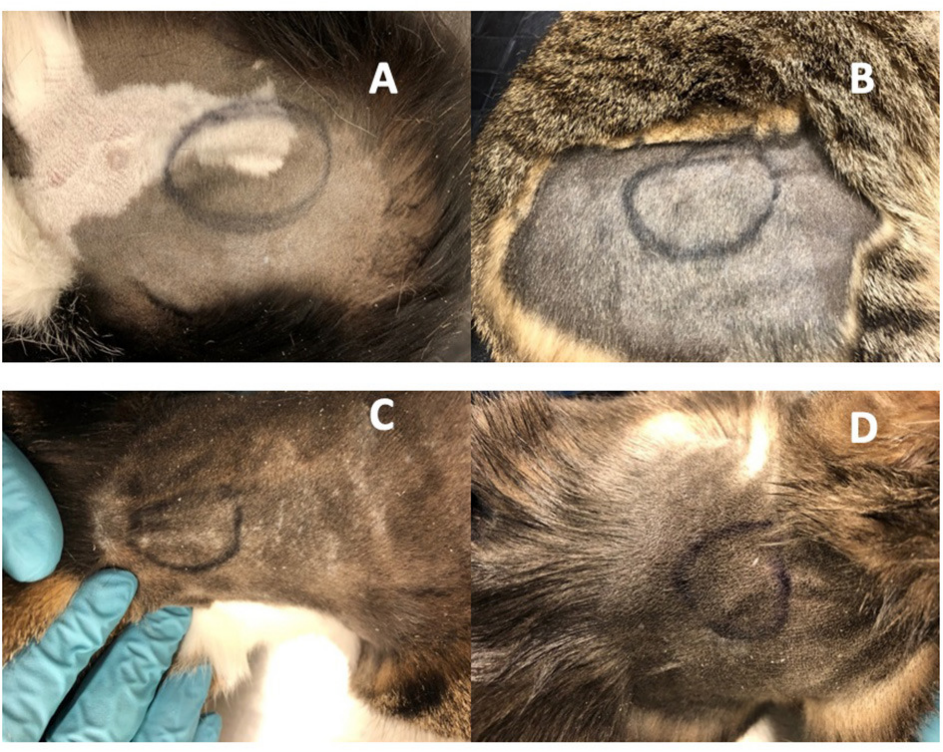
Implanted OKV-119 systems in four purpose-bred cats **(A–D)**. Images were taken on Day 61 prior to the removal procedure.

Cats appeared to tolerate the implants well. Cats did not lick or scratch the implant site and no visible evidence of inflammation was observed in the skin overlying the implant (Figure 3). There were no apparent systemic drug-related AEs clinically and no significant abnormalities on serum biochemistry and complete blood counts during the study period.

### Exenatide Plasma Concentrations and Body Weight Observations

**Figure 4:**
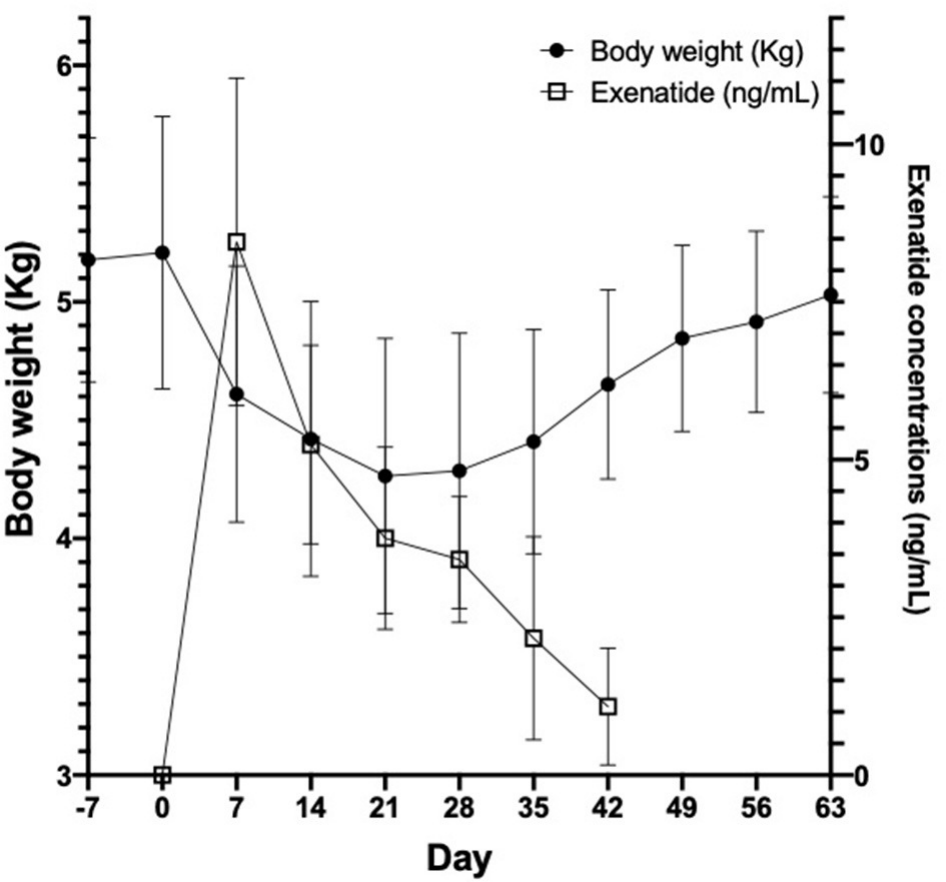
Body weight (left axis, black circles) and exenatide plasma concentrations (right axis, open squares) in 4 healthy purpose-bred cats over 9 weeks, before and after insertion of OKV-119 configured with supratherapeutic levels of exenatide. Shown are mean (±SD).

In all cats studied, plasma exenatide concentrations were below the level of detection at baseline. In the safety and tolerability study, there was no difference in body weight between day −7 and day 0 (median [range] difference = 35 g [−55 to +107 g]). Following insertion of OKV-119 systems configured to release supratherapeutic doses of exenatide, mean exenatide plasma concentrations peaked at week 1 (median [range] = 9.3 ng/mL [4.7–10.5]), and were maintained above 2 ng/mL in all cats for 28 days (Figure 4). Weight loss was observed in all four cats in the first 3 weeks (Figure 4). After the first month, body weight started to rebound when exenatide was depleted and no longer being released (Figure 4).

**Figure 5:**
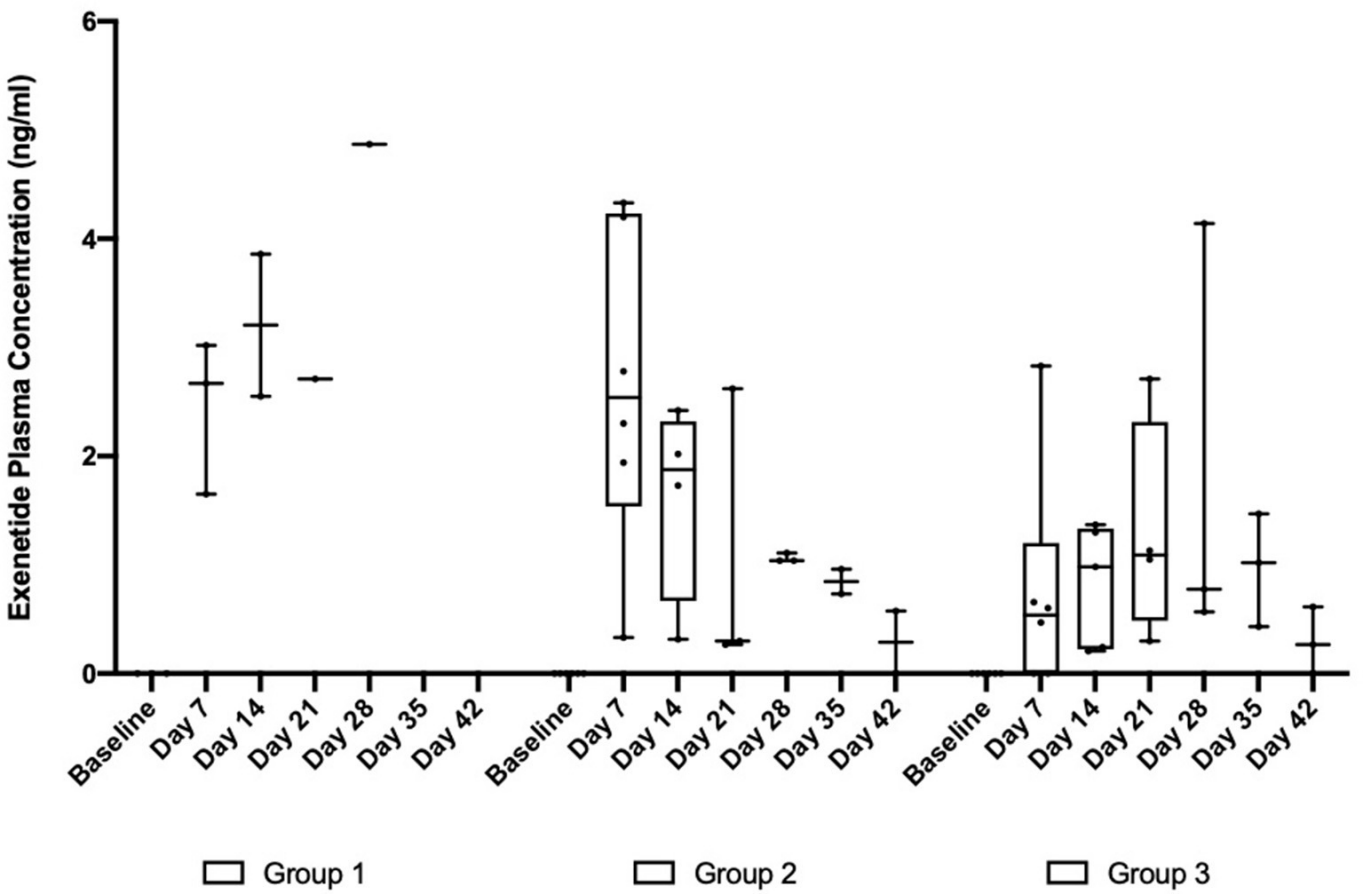
Exenatide plasma concentrations in in healthy purpose-bred cats randomized to Group 1 (*n* = 3, largest NanoPortal configuration, fast release), Group 2 (*n* = 6, medium configuration), and Group 3 (*n* = 6, thickest NanoPortal configuration, slow release). The horizontal line in each box represents the median, the lower and upper boundaries of the boxes the interquartile range, and the ends of the 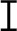 bars the minimum and maximum values.

In the subsequent OKV-119 drug-releasing concept study, average Day 7 plasma exenatide levels were 2.45 ng/ml (range: 1.65–3.02 ng/ml) and 2.65 ng/ml (range: 0.33–4.33 ng/ml) in Groups 1 and 2, respectively (Table 1 and Figure 5). In Group 3, the average Day 7 plasma exenatide levels were noticeably lower (0.76 ng/ml), and were below the level of detection in 2 out of 6 cats (Table 1 and Figure 5). Exenatide plasma concentrations were measured for up to 35 days in all three groups (Table 1 and Figure 5).

**Figure 6:**
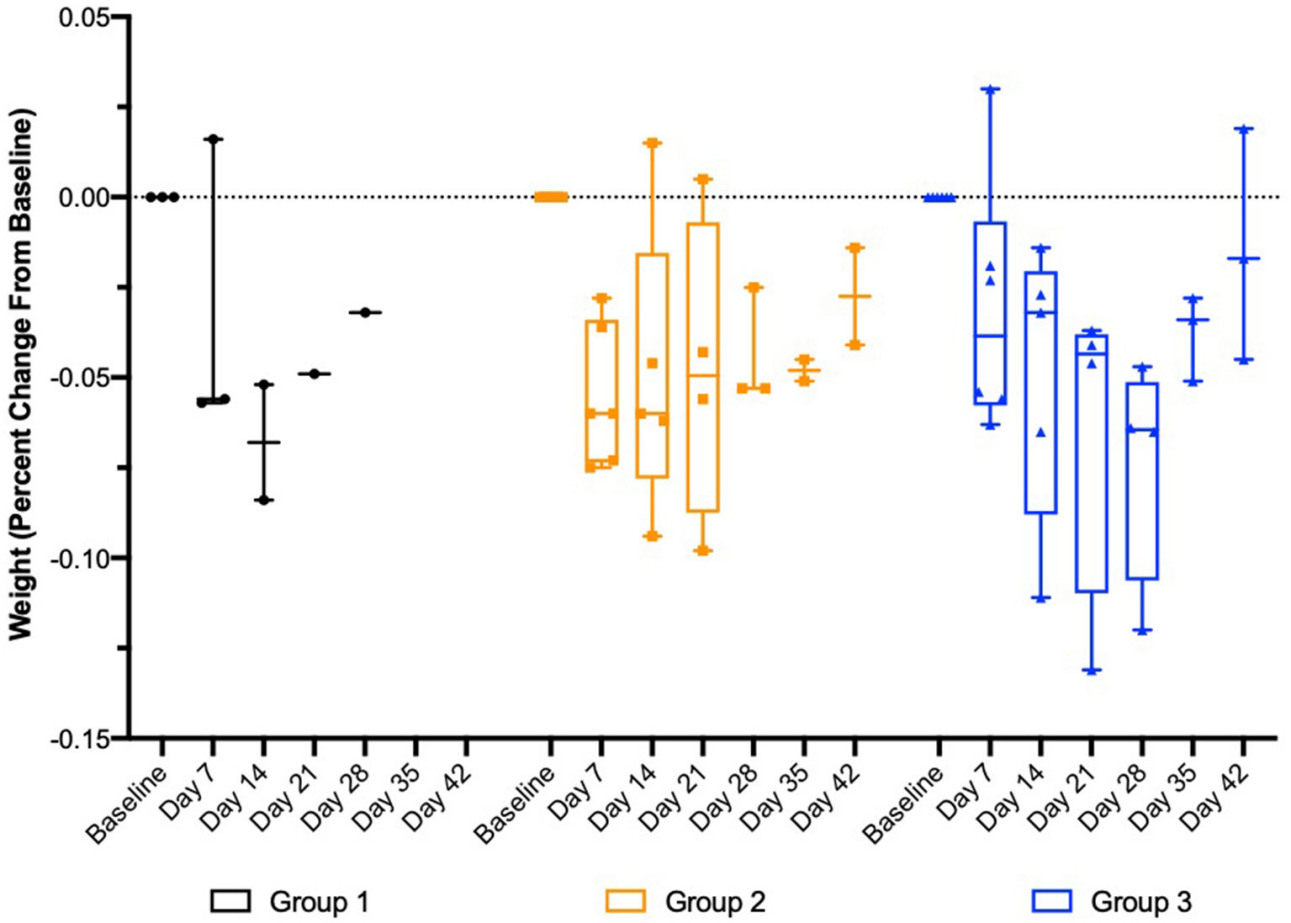
Change in body weight in healthy purpose-bred cats randomized to Group 1 (*n* = 3, largest NanoPortal configuration, fast release), Group 2 (*n* = 6, medium configuration), and Group 3 (*n* = 6, thickest NanoPortal configuration, slow release). The horizontal line in each box represents the median, the lower and upper boundaries of the boxes the interquartile range, and the ends of the 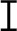 bars the minimum and maximum values.

Following implantation, weekly plasma exenatide concentrations were correlated with weight loss in the safety and tolerability study (r = −0.8 [95% CI = −0.9 to −0.6], *p* < 0.0001) and the larger study evaluating drug release of OKV-119 prototypes (−0.44 [95% CI = −0.64 to −0.18], *p* < 0.0013 after removing three outliers). Consistent weight loss was observed in all three groups (Figure 6).

## Discussion

This is the first report, in cats, of an investigational drug delivery system (OKV-119) that is under development for the treatment of feline obesity and diabetes mellitus. We present our initial experiences with the implant insertion and removal procedures, as well as preliminary tolerability and safety observations. The proof-of-concept study demonstrates that exenatide was released for a 35-day period and suggests that the OKV-119 drug delivery system is tunable and can be configured for cats. Additional study of OKV-119 implants will be needed to verify that constant target drug delivery rates can be maintained for longer, months long duration.

The subcutaneous insertion and removal of the implant in cats was found to be quick and simple, suggesting that these procedures may be performed during routine clinic visits under short anesthesia (for example with a dexmedetomidine injection that is then reversed with atipamezole). No implant-site AEs were observed, but larger and longer studies will be needed to verify that the OKV-119 implant is biocompatible and will be well-tolerated by cats over months and years. In particular, although feline injection site sarcomas are believed to be exceedingly rare and have not been linked to daily insulin injections or to microchip implants (35–37), this risk will need to be ruled out for the OKV-119 implantable system.

Feline obesity is a significant health concern with current weight management strategies largely limited to restricting caloric intake (38, 39); however, given their clinical success in managing obesity in human patients, GLP-1RAs may also have beneficial weight-loss properties in cats (14). In the initial safety and tolerability study, supratherapeutic exenatide plasma concentrations coincided with rapid and substantial weight loss in healthy cats (>10% in the first week, >15% in the first 3 weeks). In the subsequent OKV-119 concept study testing prototype delivery systems, weight loss was also correlated with exenatide plasma concentrations but with a lower (and more clinically relevant) rate of change, especially in the “slow release” group. These observations are consistent with other studies which demonstrate the weight-loss effects of GLP-1ARs in cats (14, 40–42).

Studies show that overweight people administered exenatide generally experience clinically beneficial weight loss within the first weeks of treatment, with body weight stabilizing after a few months, and then remaining relatively unchanged over years of treatment (29, 43, 44). The present study design did not enable us to capture sufficient exenatide plasma concentrations to characterize the full pharmacokinetic profiles, nor were we able to characterize target exanatide plasma concentrations required to achieve the desired weight loss in cats [e.g., 1–2% loss of body weight per week (18)]. Based on these preliminary observations, future dose-ranging studies evaluating OKV-119 systems configured to achieve exenatide plasma concentrations in the range of 1–4 ng/mL are warrented. Prospective studies of longer duration with longer exposure to exenatide in client-owned cats will also be required to establish whether, as reported in people, beneficial weight loss stabilizes, is safely maintained, and leads to metabolic improvements (7, 29, 45).

Following extended exposure to exenatide, anti-exenatide antibody formation has been detected in rats, monkeys, and humans. The presence of antibodies does not appear to have neutralizing activity in these species, although antibody titers were observed to influence pharmacokinetic parameters [NDA #22-200]. It is suspected that higher anti-drug antibody (ADA) titers may lead to an apparent increase in exposure because of the perturbation of the renal clearance of exenatide in the presence of ADA [NDA #22-200]. In future studies, anti-exenatide antibody titers will need to be measured to determine whether they have neutralizing effects in felines and/or whether they affect drug exposure.

In cats, anorexia and negative energy balance that results in excessive weight loss are clinical hallmarks of hepatic lipidosis (HL); however, the pathophysiology is incompletely understood and it remains unknown why some cats develop HL while other cats with a similar negative energy balance do not (46–48). Experimental evidence is mixed, with some laboratory cat studies showing that rapid weight-loss increases the risk of HL, while other studies suggest that gradual weight-loss is safe and is not associated with HL (49–52). In the safety study, we did not observe any clinical signs consistent with HL despite rapid weight loss. After Day 28, appetite rebounded and cat weight returned to normal as exenatide plasma concentrations declined. Similarly, no evidence of HL was observed in another study in which rapid weight loss was induced with a GLP-1RA (12). Larger studies in obese cats will be needed to determine whether the beneficial hormonal milieu conferred by excess GLP-1 activity leading to weight-loss and other metabolic improvements are outweighed by the risk of developing HL. Importantly, one of the main safety advantages of OKV-119 over ultra-long-acting injectable depot formulations of exenatide (10, 14) is the ability to remove the implant and immediately discontinue exposure to the drug in the case of excessive weight loss, HL or other AEs.

Following the classification schemes used in human medicine, diabetic cats generally suffer from a type 2-like diabetes mellitus that may be phenotypically characterized by a combination of impaired insulin action in liver, muscle, and adipose tissue (insulin resistance), and beta-cell failure (53). Given the pathophysiological similarities between human and feline T2D, incretin hormones are being evaluated as treatment options for inducing and maintaining diabetic remission in cats (9). Metabolic improvements have been observed in cats administered GLP-1RAs, but larger, prospective, well-controlled studies will be needed to characterize the clinical utility of incretin hormones for managing diabetic cats. The long-term safety profile of GLP-1RAs, administered at therapeutic drug levels, also need to be better defined.

Human clinical studies performed under real world conditions have identified lack of adherence as a significant factor associated with poorer glycemic control and other diabetes associated complications (21, 54–59). Identified barriers to adherence include cost, tolerability, and complexity and method of administration (60). Importantly, human patients prescribed injectable medications are less likely to initiate and maintain treatment compared to those taking only oral medications (23, 60). Factors affecting cat and dog owner adherence and persistence, defined as the duration of time from initiation to discontinuation of therapy, with injectable antihyperglycemics are not well-defined, but similar barriers are likely at play when people care for their pets (61).

Injection concerns (e.g., aversion to needles, needle size) are believed to negatively impact pet-owner adherence rates and may partly account for the 10% euthanasia rate reported for newly diagnosed diabetic pets (62). Treatment persistence with antihyperglycemics is an equally important determinant in the long-term prognosis of feline diabetic patients. As a surrogate measure, >40% euthanasia rates reported in diabetic cats (63, 64) may be indicative of low persistence rates of long-term injectable medications such as insulin.

To address real-world adherence and persistence challenges, OKV-119 delivery systems designed to provide continuous subcutaneous delivery of exenatide for a months-long duration are under development. Pharmacokinetic studies, in cats, using future OKV-119 prototype systems will be needed to better target *in vivo* exenatide plasma concentrations relative to OKV-119 configurations (i.e., release rate). The implant insertion and removal procedures also need to be refined before the protocols can be incorporated into routine clinic visits. Ultimately, clinical studies in client-owned obese and diabetic feline patients will be needed to evaluate both the clinical and logistical benefits of the “hassle-free” long-term delivery of exenatide.

## Data Availability Statement

The original contributions presented in the study are included in the article/supplementary material, further inquiries can be directed to the corresponding authors.

## Ethics Statement

The animal study was reviewed and approved by University of Florida Institutional Animal Care and Use Committee and by the AAALAC accredited and USDA registered testing facility Institutional Animal Care and Use Committee (IACUC).

## Author Contributions

MK, WA, and CG contributed to conception and design of the study. CA contributed to the development of the insertion and removal procedures. All authors contributed to manuscript revision, read, and approved the submitted version.

## Conflict of Interest

MK and WA are Okava Pharmaceuticals shareholders. CG is a consultant to Okava Pharmaceuticals. The remaining author declares that the research was conducted in the absence of any commercial or financial relationships that could be construed as a potential conflict of interest.

## Footnotes

^1^Alturas Analytics, Inc., 1324 Alturas Drive, Moscow, ID 83843.

^2^GraphPad Prism, version 8.0; GraphPad Software Inc, La Jolla, CA.

## Abbreviations

AE: Adverse events
ER: Extended-release
GLP-1: Glucagon-like peptide-1
GLP-1RA: GLP-1 receptor agonists
HL: Hepatic lipidosis.

## References

[1] Lorenz M Evers A Wagner M. Recent progress and future options in the development of GLP-1 receptor agonists for the treatment of diabesity. Bioorg Med Chem Lett. (2013) 23:4011–8. 10.1016/j.bmcl.2013.05.022 23743288

[2] Lyseng-Williamson KA. Glucagon-like peptide-1 receptor analogues in type 2 diabetes: their use and differential features. Clin Drug Investig. (2019) 39:805–19. 10.1007/s40261-019-00826-0 31317516 PMC6746674

[3] Byetta [Package Insert].Wilmington, DE: AstraZeneca Pharmaceuticals, LP (2018).

[4] Su N Li Y Xu T Li L Kwong JS Du H. Exenatide in obese or overweight patients without diabetes: a systematic review and meta-analyses of randomized controlled trials. Int J Cardiol. (2016) 219:293–300. 10.1016/j.ijcard.2016.06.028 27343423

[5] Domecq JP Prutsky G Leppin A Sonbol MB Altayar O Undavalli C. Clinical review: drugs commonly associated with weight change: a systematic review and meta-analysis. J Clin Endocrinol Metab. (2015) 100:363–70. 10.1210/jc.2014-3421 25590213 PMC5393509

[6] Victoza [Package Insert]. Plainsboro, NJ: Novo Nordisk Inc. (2019).

[7] Wilding JPH Batterham RL Calanna S Davies M Van Gaal LF Lingvay I. Once-weekly semaglutide in adults with overweight or obesity. N Engl J Med. (2021) 384:989–1002. 10.1056/NEJMoa2032183 33567185

[8] Verdich C Toubro S Buemann B Lysgård Madsen J Juul Holst J Astrup A. The role of postprandial releases of insulin and incretin hormones in meal-induced satiety–effect of obesity and weight reduction. Int J Obes Relat Metab Disord. (2001) 25:1206–14. 10.1038/sj.ijo.0801655 11477506

[9] Gilor C Rudinsky AJ Hall MJ. New approaches to feline diabetes mellitus: glucagon-like peptide-1 analogs. J Feline Med Surg. (2016) 18:733–43. 10.1177/1098612X16660441 27562982 PMC11148896

[10] Rudinsky AJ Adin CA Borin-Crivellenti S Rajala-Schultz P Hall MJ Gilor C. Pharmacology of the glucagon-like peptide-1 analog exenatide extended-release in healthy cats. Domest Anim Endocrinol. (2015) 51:78–85. 10.1016/j.domaniend.2014.12.003 25594949

[11] Gilor C Graves TK Gilor S Ridge TK Rick M. The GLP-1 mimetic exenatide potentiates insulin secretion in healthy cats. Domest Anim Endocrinol. (2011) 41:42–9. 10.1016/j.domaniend.2011.03.001 21645806

[12] Hall MJ Adin CA Borin-Crivellenti S Rudinsky AJ Rajala-Schultz P Lakritz J. Pharmacokinetics and pharmacodynamics of the glucagon-like peptide-1 analog liraglutide in healthy cats. Domest Anim Endocrinol. (2015) 51:114–21. 10.1016/j.domaniend.2014.12.001 25625650

[13] Hoelmkjaer KM Wewer Albrechtsen NJ Holst JJ Cronin AM Nielsen DH Mandrup-Poulsen T. A placebo-controlled study on the effects of the glucagon-like peptide-1 mimetic, exenatide, on insulin secretion, body composition and adipokines in obese, client-owned cats. PLoS ONE. (2016) 11:e0154727. 10.1371/journal.pone.0154727 27136422 PMC4852899

[14] Schneider EL Reid R Parkes DG Lutz TA Ashley GW Santi DV. A once-monthly GLP-1 receptor agonist for treatment of diabetic cats. Domest Anim Endocrinol. (2019) 70:106373. 10.1016/j.domaniend.2019.07.001 31479925

[15] Husnik R Gaschen FP Fletcher JM Gaschen L. Ultrasonographic assessment of the effect of metoclopramide, erythromycin, and exenatide on solid-phase gastric emptying in healthy cats. J Vet Intern Med. (2020) 34:1440–446. 10.1111/jvim.15787 32515089 PMC7379023

[16] Riederer A Zini E Salesov E Fracassi F Padrutt I Macha K. Effect of the glucagon-like peptide-1 analogue exenatide extended release in cats with newly diagnosed diabetes mellitus. J Vet Intern Med. (2016) 30:92–100. 10.1111/jvim.13817 26700409 PMC4913624

[17] Day MJ. One health approach to preventing obesity in people and their pets. J Compar Pathol. (2017) 156:293–5. 10.1016/j.jcpa.2017.03.009 28457487

[18] Flanagan J Bissot T Hours MA Moreno B German AJ. An international multi-centre cohort study of weight loss in overweight cats: differences in outcome in different geographical locations. PLoS ONE. (2018) 13:e0200414. 10.1371/journal.pone.0200414 30044843 PMC6059437

[19] Chandler M Cunningham S Lund EM Khanna C Naramore R Patel A. Obesity and associated comorbidities in people and companion animals: a one health perspective. J Comp Pathol. (2017) 156:296–309. 10.1016/j.jcpa.2017.03.006 28460795

[20] Zoran DL. Obesity in dogs and cats: a metabolic and endocrine disorder. Vet Clin North Am Small Anim Pract. (2010) 40:221–39. 10.1016/j.cvsm.2009.10.009 20219485

[21] Giorgino F Penfornis A Pechtner V Gentilella R Corcos A. Adherence to antihyperglycemic medications and glucagon-like peptide 1-receptor agonists in type 2 diabetes: clinical consequences and strategies for improvement. Patient Prefer Adherence. (2018) 12:707–19. 10.2147/PPA.S151736 29765207 PMC5944456

[22] Carls GS Tuttle E Tan RD Huynh J Yee J Edelman SV. Understanding the gap between efficacy in randomized controlled trials and effectiveness in real-world use of GLP-1 RA and DPP-4 therapies in patients with type 2 diabetes. Diabetes Care. (2017) 40:1469–78. 10.2337/dc16-2725 28801475

[23] Spain CV Wright JJ Hahn RM Wivel A Martin AA. Self-reported barriers to adherence and persistence to treatment with injectable medications for type 2 diabetes. Clin Ther. (2016) 38:1653–64.e1. 10.1016/j.clinthera.2016.05.009 27364806

[24] Rubin RR Peyrot M Kruger DF Travis LB. Barriers to insulin injection therapy: patient and health care provider perspectives. Diabetes Educ. (2009) 35:1014–22. 10.1177/0145721709345773 19934459

[25] Polonsky WH Henry RR. Poor medication adherence in type 2 diabetes: recognizing the scope of the problem and its key contributors. Patient Prefer Adherence. (2016) 10:1299–307. 10.2147/PPA.S106821 27524885 PMC4966497

[26] Mody R Huang Q Yu M Zhao R Patel H Grabner M. Adherence, persistence, glycaemic control and costs among patients with type 2 diabetes initiating dulaglutide compared with liraglutide or exenatide once weekly at 12-month follow-up in a real-world setting in the United States. Diabetes Obesity Metab. (2019) 21:920–9. 10.1111/dom.13603 30520248 PMC6590811

[27] Restine LM Norsworthy GD Kass PH. Loose-control of diabetes mellitus with protamine zinc insulin in cats: 185 cases (2005-2015). Can Vet J. (2019) 60:399–404. 30992596 PMC6417615

[28] Henry R Rosenstock J McCarthy JF Carls G Alessi T Yee J. Treatment satisfaction with ITCA 650, a novel drug-device delivering continuous exenatide, versus twice-daily injections of exenatide in type 2 diabetics using metformin. Diabetes Obes Metab. (2018) 20:638–45. 10.1111/dom.13133 29053202

[29] Henry RR Rosenstock J Logan D Alessi T Luskey K Baron MA. Continuous subcutaneous delivery of exenatide via ITCA 650 leads to sustained glycemic control and weight loss for 48 weeks in metformin-treated subjects with type 2 diabetes. J Diabetes Complicat. (2014) 28:393–8. 10.1016/j.jdiacomp.2013.12.009 24631129

[30] Siven M Savolainen S Rantila S Mannikko S Vainionpaa M Airaksinen S. Difficulties in administration of oral medication formulations to pet cats: an e-survey of cat owners. Vet Rec. (2017) 180:250. 10.1136/vr.103991 27980080

[31] Hullar I Fekete S Andrasofszky E Szocs Z Berkenyi T. Factors influencing the food preference of cats. J Anim Physiol Anim Nutr. (2001) 85:205–11. 10.1046/j.1439-0396.2001.00333.x 11686790

[32] Amiram M Luginbuhl KM Li X Feinglos MN Chilkoti A. Injectable protease-operated depots of glucagon-like peptide-1 provide extended and tunable glucose control. Proc Natl Acad Sci USA. (2013) 110:2792–7. 10.1073/pnas.1214518110 23359691 PMC3581961

[33] Fischer KDK Fischer W Mendelsohn A. Nanoscale constrained delivery: a novel technology for subdermal implants. CRS Newsletters. 31 (2014).

[34] Fischer K Duong Y Fischer W Mendelsohn A. A Pure Titanium/Titania Implant for Tunable Zero-Order Drug Delivery. (2014). Available online at: http://www.nanoprecisionmedical.com/pdfs/publications/crs-poster-2014.pdf

[35] Dean RS Pfeiffer DU Adams VJ. The incidence of feline injection site sarcomas in the United Kingdom. BMC Vet Res. (2013) 9:17. 10.1186/1746-6148-9-17 23339769 PMC3608079

[36] Martano M Morello E Buracco P. Feline injection-site sarcoma: past, present and future perspectives. Vet J. (2011) 188:136–41. 10.1016/j.tvjl.2010.04.025 20510635

[37] Saba CF. Vaccine-associated feline sarcoma: current perspectives. Vet Med. (2017) 8:13–20. 10.2147/VMRR.S116556 30050850 PMC6042530

[38] Larsen JA. Risk of obesity in the neutered cat. J Feline Med Surg. (2017) 19:779–83. 10.1177/1098612X16660605 27432438 PMC11104111

[39] Loftus JP Wakshlag JJ. Canine and feline obesity: a review of pathophysiology, epidemiology, clinical management. Vet Med. (2015) 6:49–60. 10.2147/VMRR.S40868 30101096 PMC6067794

[40] Aroda VR. A review of GLP-1 receptor agonists: evolution and advancement, through the lens of randomised controlled trials. Diabetes Obes Metab. (2018) 20 (Suppl. 1):22–33. 10.1111/dom.13162 29364586

[41] Scuderi MA Ribeiro Petito M Unniappan S Waldner C Mehain S McMillian CJ. Safety and efficacy assessment of a GLP-1 mimetic: insulin glargine combination for treatment of feline diabetes mellitus. Domest Anim Endocrinol. (2018) 65:80–9. 10.1016/j.domaniend.2018.04.003 30015124

[42] Muller TD Finan B Bloom SR D'Alessio D Drucker DJ Flatt PR. Glucagon-like peptide 1 (GLP-1). Mol Metab. (2019) 30:72–130. 10.1016/j.molmet.2019.09.010 31767182 PMC6812410

[43] Diamant M Van Gaal L Guerci B Stranks S Han J Malloy J. Exenatide once weekly versus insulin glargine for type 2 diabetes (DURATION-3):3-year results of an open-label randomised trial. Lancet Diabetes Endocrinol. (2014) 2:464–73. 10.1016/S2213-8587(14)70029-4 24731672

[44] Buse JB Drucker DJ Taylor KL Kim T Walsh B Hu H. DURATION-1: exenatide once weekly produces sustained glycemic control and weight loss over 52 weeks. Diabetes Care. (2010) 33:1255. 10.2337/dc09-1914 20215461 PMC2875434

[45] Rosenstock J Buse JB Azeem R Prabhakar P Kjems L Huang H. Efficacy and safety of ITCA 650, a novel drug-device GLP-1 receptor agonist, in type 2 diabetes uncontrolled with oral antidiabetes drugs: the FREEDOM-1 trial. Diabetes Care. (2018) 41:333–40. 10.2337/dc17-1306 29242349

[46] Webb CB. Hepatic lipidosis: clinical review drawn from collective effort. J Feline Med Surg. (2018) 20:217–27. 10.1177/1098612X18758591 29478399 PMC10816292

[47] Valtolina C Favier RP. Feline hepatic lipidosis. Vet Clin North Am Small Anim Pract. (2017) 47:683–702. 10.1016/j.cvsm.2016.11.014 28108035

[48] Kuzi S Segev G Kedar S Yas E Aroch I. Prognostic markers in feline hepatic lipidosis: a retrospective study of 71 cats. Vet Rec. (2017) 181:512. 10.1136/vr.104252 28978714

[49] Biourge V Pion P Lewis J Morris JG Rogers QR. Spontaneous occurrence of hepatic lipidosis in a group of laboratory cats. J Vet Intern Med. (1993) 7:194–7. 10.1111/j.1939-1676.1993.tb03186.x 8331615

[50] Biourge VC Groff JM Munn RJ Kirk CA Nyland TG Madeiros VA. Experimental induction of hepatic lipidosis in cats. Am J Vet Res. (1994) 55:1291–302. 7802398

[51] Szabo J Ibrahim WH Sunvold GD Dickey KM Rodgers JB Toth IE. Influence of dietary protein and lipid on weight loss in obese ovariohysterectomized cats. Am J Vet Res. (2000) 61:559–65. 10.2460/ajvr.2000.61.559 10803653

[52] Dimski DS Buffington C Johnson SE. Serum lipoprotein concentrations and hepatic lesions in obese cats undergoing weight loss. Am J Vet Res. (1992) 53:1259–62. 1497199

[53] Nelson RW Reusch CE. Animal models of disease: classification and etiology of diabetes in dogs and cats. J Endocrinol. (2014) 222:T1–9. 10.1530/JOE-14-0202 24982466

[54] Hamersky CM Fridman M Gamble CL Iyer NN. Injectable antihyperglycemics: a systematic review and critical analysis of the literature on adherence, persistence, health outcomes. Diabetes Ther. (2019) 10:865–90. 10.1007/s13300-019-0617-3 31054132 PMC6531561

[55] Asche C LaFleur J Conner C. A review of diabetes treatment adherence and the association with clinical and economic outcomes. Clin Ther. (2011) 33:74–109. 10.1016/j.clinthera.2011.01.019 21397776

[56] Guerci B Chanan N Kaur S Jasso-Mosqueda JG Lew E. Lack of treatment persistence and treatment nonadherence as barriers to glycaemic control in patients with type 2 diabetes. Diabetes Ther. (2019) 10:437–49. 10.1007/s13300-019-0590-x 30850934 PMC6437240

[57] Kim Y-Y Lee J-S Kang H-J Park SM. Effect of medication adherence on long-term all-cause-mortality and hospitalization for cardiovascular disease in 65,067 newly diagnosed type 2 diabetes patients. Sci Rep. (2018) 8:12190. 10.1038/s41598-018-30740-y 30111867 PMC6093904

[58] Perez-Nieves M Boye KS Kiljanski J Cao D Lage MJ. Adherence to basal insulin therapy among people with type 2 diabetes: a retrospective cohort study of costs and patient outcomes. Diabetes Ther. (2018) 9:1099–111. 10.1007/s13300-018-0421-5 29644618 PMC5984924

[59] Carls GS Tan R Zhu JY Tuttle E Yee J Edelman SV. Real-world weight change among patients treated with glucagon-like peptide-1 receptor agonist, dipeptidyl peptidase-4 inhibitor and sulfonylureas for type 2 diabetes and the influence of medication adherence. Obes Sci Pract. (2017) 3:342–51. 10.1002/osp4.116 29071110 PMC5598021

[60] Peyrot M Barnett AH Meneghini LF Schumm-Draeger PM. Insulin adherence behaviours and barriers in the multinational Global Attitudes of Patients and Physicians in Insulin Therapy study. Diabet Med. (2012) 29:682–9. 10.1111/j.1464-5491.2012.03605.x 22313123 PMC3433794

[61] Wareham KJ Brennan ML Dean RS. Systematic review of the factors affecting cat and dog owner compliance with pharmaceutical treatment recommendations. Vet Rec. (2019) 184:154. 10.1136/vr.104793 30455188

[62] Niessen SJM Hazuchova K Powney SL Guitian J Niessen APM Pion PD. The big pet diabetes survey: perceived frequency and triggers for euthanasia. Vet Sci. (2017) 4:27. 10.3390/vetsci4020027 29056686 PMC5606606

[63] O'Neill DG Gostelow R Orme C Church DB Niessen SJ Verheyen K. Epidemiology of diabetes mellitus among 193,435 cats attending primary-care veterinary practices in England. J Vet Intern Med. (2016) 30:964–72. 10.1111/jvim.14365 27353396 PMC5094533

[64] Callegari C Mercuriali E Hafner M Coppola LM Guazzetti S Lutz TA. Survival time and prognostic factors in cats with newly diagnosed diabetes mellitus: 114 cases (2000-2009). J Am Vet Med Assoc. (2013) 243:91–5. 10.2460/javma.243.1.91 23786195

